# Long-Term Drug Misuse Increases the Risk of Cognitive Dysfunctions in Intimate Partner Violence Perpetrators: Key Intervention Targets for Reducing Dropout and Reoffending

**DOI:** 10.3390/ijerph16203792

**Published:** 2019-10-09

**Authors:** Ángel Romero-Martínez, Marisol Lila, Luis Moya-Albiol

**Affiliations:** 1 Department of Psychobiology, University of Valencia, Avenida Blasco, Ibañez, 21 46010 Valencia, Spain; Luis.Moya@uv.es; 2 Department of Social Psychology, University of Valencia, Avenida Blasco, Ibañez, 21 46010 Valencia, Spain; Marisol.Lila@uv.es

**Keywords:** cognition, decision-making process, drug misuse, intimate partner violence against women, reoffending

## Abstract

Intimate partner violence against women (IPVAW) is a major public health problem, with an important mortality rate in women across the world. In this regard, it has been well-established that drug misuse explains (at least in part) an increased risk of IPVAW perpetration. Even though alcohol is the most widely studied drug underlying IPVAW, other drugs, such as cannabis and cocaine also seem to be significant indicators of this type of violence. Nonetheless, little is known about mediators, such as cognitive domains that facilitate proneness to violence after drug consumption. Therefore, the primary objective of the present study was to compare drug misuse patterns and cognitive performance in a carefully selected sample of IPVAW perpetrators (*n* = 63) and a group of non-violent men (control group; *n* = 39). Second, we also aimed to study the association between different patterns of drug misuse and cognitive performance and several facets of IPVAW perpetration (i.e., severity of injuries and type of aggression). Our results revealed that IPVAW perpetrators showed considerably higher levels of sustained drug misuse (alcohol, cannabis, cocaine, and heroin) for years and worse cognitive performance than controls. Moreover, the highest drug misuse sustained over time was related to the worst cognitive performance and the highest IPVAW severity. Finally, alcohol and cocaine seemed to be related to IPVAW and risk of reoffending. Whereas, cannabis, heroin, and MDMA were related to the existence of a previous criminal record (delinquency without violence). Hence, research in this field would help to develop coadjutant treatments and intervention packages to reduce drug misuse in the initial stages, which in turn would reduce cognitive impairments in IPVAW perpetrators. These expected improvements might produce an increase in treatment adherence and a decrease in the risk of future IPVAW reoffending.

## 1. Introduction

Intimate partner violence against women (IPVAW) is a major public health problem, with severe consequences for victims’ physical and mental well-being, and an important cause in women’s mortality rate across the world [1,2,3,4]. In addition, it has been well-established that drug misuse explains (at least in part) the increased risk of IPVAW perpetration. In fact, it is well-established that IPVAW is more common among individuals who use drugs, being this risk even greater when both partners (aggressor and victim) are drug consumers. In this sense, there is a vast body of scientific literature that considers alcohol as a good predictor of IPVAW perpetration and/or maintenance [5,6,7]. However, this does not discard the possibility that other drugs, such as cannabis and cocaine, may also be significant indicators of this type of violence [5,6,7]. Nevertheless, the ways in which drug misuse might increase proneness to violence is less obvious. Several hypotheses have tried to explain its facilitator role. Thus, violence has been explained by intoxication, due to drug effects, or by behavioral dysregulations from drug misuse over a long period of time [5,8,9,10,11,12]. Nevertheless, it is necessary to closely examine specific variables that are closely related to behavioral regulation, and that are especially sensitive to drug misuse, in order to understand the complex phenomenon of IPVAW.

Most studies have concluded that alcohol is a robust indicator of IPVAW [5,13,14,15]. In fact, it has been demonstrated that there are three main ways to explain its IPVAW facilitator role. First, after acute alcohol intoxication, individuals tend to experience a deceleration of cognitive and emotional processes, as well as a restricted ability to process the most salient external stimuli [15,16]. Moreover, if the individual has hostile cognitive schemas, high trait anger, and/or personality disorders, among others, along with the previously mentioned restricted ability to process information during intoxicated periods, alcohol would facilitate violent reactions, even in neutral contexts [11,12,17]. 

Regarding the second hypothesis, violent reactions in drug misusers have been explained by craving (or the desire to obtain this substance) during alcohol withdrawal, whose brain correlate is limbic system irritability. This has been defined as difficulties associated with the control of temporo-limbic activation by prefrontal brain structures when faced with certain types of stimulus or conflict contexts. Some of their behavioral correlates include anxiety and hostility, among others [18]. The third hypothesis, which complements the other two, involves cognitive impairments stemming from heavy alcohol consumption over long periods of time (even years). Individuals who present a sustained and heavy alcohol use tend to show alterations in executive functioning, which might mean that they present difficulties in regulating their emotions and behavior, without making realistic plans and experiencing alterations in decision-making processes. Hence, these individuals tend to respond with violence, in order to cope with stress or stressful conditions [10,19,20,21]. 

Other illicit substances, apart from alcohol, have also been considered robust indicators of IPVAW. In this regard, systematic reviews have highlighted the role of psychostimulants, such as cocaine and MDMA (or ecstasy) as important contributors to IPVAW perpetration in interactions with specific personality traits and/or other contextual factors [7,11,12,22,23,24,25]. Initially, a possible explanation for their facilitation role is the hyperexcitation of the limbic reward system after their consumption, which tends to lead to symptoms, such as high irritability, aggressiveness, and paranoia, among others [11,12,26]. Furthermore, the person is highly likely to experience increased irritability due to cravings during psychostimulant withdrawal or a worsening of comorbid psychiatric symptoms [11,12,26,27]. Finally, heavy cocaine consumption for years might lead to different cognitive and empathic deficits that impair behavioral and emotional regulation [11,12]. Moreover, the interactions between psychostimulants and other drugs, such as alcohol, exponentially increase their toxic effects [11,12]. Nevertheless, little is known about whether IPVAW perpetrators tend to present cognitive impairments, due to heavy and sustained cocaine misuse.

Although, we previously presented the illicit drugs that are robustly associated with IPVAW, cannabis, heroin, and anabolic steroids, seem to show a bidirectional relationship with violence [11,12,24,28]. As in the previously mentioned drugs, the facilitation of violence seems to be explained by intoxication effects as a result of craving during withdrawal and/or behavioral dysregulations, due to heavy and sustained use of these drugs over time [7,29]. Additionally, the misuse of these drugs interacting with individual hostile traits and/or biases (e.g., personal traits, psychopathological symptoms, brain injuries, and polydrug use), which facilitate IPVAW or other types of violence, when faced with adequate stimulation. However, as far as we know, there is a gap in the scientific literature in analyzing the main cognitive impairments after a sustained period of drug misuse and the way these deficits are related to IPVAW perpetration.

In accordance with the aforementioned studies, the primary objective of the present study was to compare the drug misuse patterns and cognitive performance of a carefully selected sample of IPVAW perpetrators and a group of participants who served as a control group (non-IPVAW). Thus, as previously established [5,6,7,10], we hypothesized that the highest drug misuse and the worst cognitive performance would be presented by IPVAW perpetrators. Moreover, we were also interested in analyzing the association between drug misuse patterns (weekly drug summary and number of consecutive years consuming), and cognitive performance in both groups and IPVAW perpetration (only male abusers). Therefore, based on the main results in this field of research [7,11,12,24,25,28,29], we would expect to find that high and sustained drug misuse over years would be associated with worse cognitive performance. Furthermore, the higher the drug misuse, the greater the IPVAW severity. Hence, research on this topic would help to develop coadjutant treatments and intervention packages to improve cognitive impairments in IPVAW perpetrators. These expected improvements would produce a decrease in the risk of future IPVAW reoffending. We expect that, after cognitive training, IPVAW perpetrators would attempt to focus on the big picture, without distractions by salient external stimuli.

## 2. Method

### 2.1. Participants

The final sample of 102 participants was composed of 63 IPVAW perpetrators and 39 controls (with no criminal history of IPVAW). We ensured that all participants had a good grasp of Spanish and were able to express themselves in the language in their first interviews. Male abusers were recruited from the participants in the CONTEXTO psycho-educational and community-based treatment program of the Faculty of Psychology (University of Valencia). This intervention program receives approximately 120 men convicted of IPVAW each year. This program provides a cognitive behavioral treatment, which also includes motivational strategies, in order to increase treatment compliance and effectivity that will help promote the change. These men were sentenced to less than two years in prison (from 6 to 24 months), but their sentences were suspended on the condition that they attend this intervention program. Moreover, in order to receive this intervention program, they could not have a previous criminal record [30,31,32]. However, those participants with severe mental disorders or cognitive impairments (i.e., brain damage due to traumatic brain injuries, strokes, and schizophrenia), psychic or psychopathological problems, or drug use disorders, that interfere with the functioning of the intervention, would be excluded from the program. In order to detect these disorders, all the participants were interviewed by two independent trained researchers. They conducted two qualitative interviews based on the psychopathological and personality dimensions evaluated by the Symptom Checklist-90-Revised (SCL-90-R), and the Millon Clinical Multiaxial Inventory-III (MCMI-III), respectively. Their degree of agreement on the different psychopathological symptoms and personality disorders ranged from 0.70 to 0.90 (Cohen’s kappa). These interviews were completed with objective measurements, such as self-reports. 

In the case of the control group, they were recruited via internet advertisements and by posting flyers around our city from January 2017 to December 2017. The inclusion criteria were as follows: Males over 18 years of age with no physical or mental problems, no severe substance abuse problems, and no severe cognitive impairment. We ensured that they did not present previous criminal records by asking for an official document that would justify that.

Finally, all participants gave their written informed consent for inclusion, before they participated in the study. Furthermore, this study was conducted following the Declaration of Helsinki and approved by the University of Valencia Ethics Committee (H1348835571691).

### 2.2. Procedure 

IPVAW perpetrators were initially informed that, if they refused to participate in our study, their legal situation would not be affected. Moreover, they were also informed that the judicial system would not have access to individual responses or results provided in the research. 

Each subject participated in two sessions in the psychobiology laboratories of the University of Valencia. In the first session, the participants were interviewed to exclude any individuals with severe physical or mental illnesses that would interfere with the intervention and would have to be resolved before their participation. Moreover, they were asked about their drug use (i.e., alcohol, cannabis, and cocaine) in terms of the frequency of consumption and the amount consumed. Additionally, this interview included questions about the IPVAW perpetration (i.e., type of aggression and severity of injuries), as well as the risk of reoffending assessed by the Spousal Assault Risk Assessment (SARA). This interview was designed by trained researchers based on their expertise in this field. The second session took place on the following day between 10 am and 2 pm, in order to minimize the possible effects of fatigue later in the day. During this session, cognitive domains, such as working memory, attention, and executive functioning were assessed with pen and pencil and computerized neuropsychological tests. Each session lasted approximately 90 min, and the sessions were conducted by two researchers with a practical background in neuropsychological assessment. After the assessment ended, we thanked them for their participation and also provided them each with 20€. 

### 2.3. Neuropsychological Tests

#### 2.3.1. Memory

We employed the Letter-Number Sequencing test as a measure of working memory. Initially, the participants listened to a list of a mixture letters and numbers, and they had to first repeat the numbers in ascending order, and then the letters in alphabetical order (e.g., 8-D-1-B; correct response is 1-8-B-D) [33].

To measure visuo-spatial memory, we employed the Spatial Span (a subscale of the WMS-III). On this test, participants have to copy a series of movements, that were previously performed by the evaluator. The level of difficulty increases after each movement, with more digits in each part. Moreover, during the initial stages of this test, participants have to repeat the movements in the direct order, but in the second part, they have to repeat them in an inverse order [34].

#### 2.3.2. Attention 

To measure the sustained attention, the Rapid Visual Information Processing (RVP) test was employed. On the center of the computer screen, randomized series of three digits appear (from 2 to 9). Participants are asked to detect specific target sequences of three consecutive digits (e.g., 2,4,6; 3,5,7; and 4,6,8). The percentage of correct responses was employed as a dependent variable [35]. 

To measure the ability to switch attention, the Attention Switching Task (ATS) was used. In this test, participants had to switch their attention between congruent and incongruent stimuli (e.g., arrow on the right side of the screen pointing to the right, or left, respectively). The dependent variables for this study were congruency, switch cost, and the percentage of correct responses [35].

#### 2.3.3. Executive Functioning 

As a measure of planning ability (strategies to correctly solve a problem), we employed the Key test, which is a subtest of the Behavioral Assessment of Dysexecutive Syndrome [36]. In this test, participants have to design a trajectory to find a key lost in a specific, empty rectangle on the paper. Dependent variables for this study were total time (to plan and execute) and total score.

As a measure of cognitive flexibility, we employed the Wisconsin Card Sorting Test (WCST). This test consists of a series of 4 stimulus cards and 128 response cards containing various colors (red, blue, yellow, or green), shapes (circle, cross, star, or triangle), and numbers (one, two, three, or four) of figures [37]. Participants have to match the response cards to one of the stimulus cards, with the researcher providing feedback about their responses (e.g., correct or incorrect). The dependent variables in our study were total trials, total errors, perseverative mistakes, and completed categories [37]. 

To assess decision-making, the Cambridge Gambling Task was employed. During this test, a row of ten boxes appear across the top of the screen, some red and some blue. At the bottom of the screen, there are rectangles, containing the words “Red” and “Blue”. Participants have to decide if a yellow token is hidden in a red box or a blue box. Moreover, before making this decision, they have to gamble a proportion of points, sometimes in an increasing order and others in descending order. They must also accumulate as many points as possible during the test in order to minimize their losses [35].

#### 2.3.4. Risk of Reoffending and Intimate Partner Violence Perpetration Assessment

The Spanish version of the Spousal Assault Risk Assessment Guide (SARA) [38] was employed to assess the risk of reoffending (general violence and intimate partner violence against women). This test includes 20 items, rated on a 3-point scale (from 0 = low to 2 = high risk). The items measure: Current relationship problems, current psychotic and/or manic symptoms, or behavioral instability, among others. Moreover, it should be noted that this test was administered by trained researchers in this field. Finally, we considered the sum of the risk factors in this study. Its score ranged from 0 (absence of risk of reoffending) to 40 (imminent risk of reoffending) [38,39]. Cronbach’s alpha for this study was 0.80.

We designed an interview that included questions about the IPVAW perpetration, such as type of aggression, severity of injuries, and previous criminal history. Participants were asked these questions directly (i.e., type of aggressions, victims of their attacks, frequency of violent outburst, and previous/current antisocial behaviors). We also obtained official records from the Spanish Government, in order to check the veracity of participants’ responses (i.e., criminal history, length of prison stays). These variables were dummy coded as 0 (low risk/severity) and 1 (high risk/severity) for this study. 

### 2.4. Data Analysis

The Shapiro-Wilks test was used to explore whether the data were normally distributed. As most of the variables did not meet the assumption of normality (*p* < 0.05), we decided to carry out nonparametric tests for the statistical analysis of the results. The Mann–Whitney U test was used to check for significant differences between the groups on socio-demographic data, questionnaire scores, and neuropsychological tests. In addition, chi square analyses were performed for categorical variables, such as socio-demographic characteristics.

Pearson’s or Spearman correlation coefficients were calculated to assess the relationships between variables, where appropriate, for all the participants. Afterwards, linear regression models of these significant relationships were constructed to investigate whether the drug misuse indicators predicted cognitive performance (dependent variable) in each group (moderator variable). We ran a hierarchical regression analysis with cognitive performance as the dependent variable and the following variables as predictors: ‘Group’ in Step 1 (dummy-coded as 0 for IPVAW perpetrators and 1 for controls); drug misuse (weekly amount and number of years consuming) in Step 2; and the two-way interactions (i.e., Group x drug misuse variable) in Step 3. Finally, when a significant two-way interaction (Step 3) was found, we ran separate regression models in IPVAW perpetrators and controls. 

Finally, Pearson’s or Spearman correlation coefficients were calculated to assess the relationships between drug misuse and IPVAW-related variables (type of violence, severity of injuries, and a previous criminal record not related to violence), only in the IPVAW perpetrator group. In order to reduce the risk of obtaining false positives (type I errors), we applied a Bonferroni correction for multiple comparisons.

Data analyses were carried out using IBM SPSS Statistics for Windows, Version 24.0 (IBM, Armonk, NY, USA). *p* values *<* 0.05 were considered statistically significant. Average values are reported in tables as mean ± SD.

## 3. Results

The descriptive characteristics for the IPVAW group and controls are presented in Table 1. Although, there were no differences found between groups on the demographic variables (e.g., age, educational level, and income), IPVAW perpetrators showed higher drug misuse, specifically alcohol, cannabis, cocaine, and heroin, than the control group (*p* < 0.05). Nevertheless, there were no differences between groups in MDMA and anabolic steroid consumption.

Regarding the risk of reoffending and IPVAW perpetration assessment, 67% of IPVAW perpetrators presented physical and psychological IPVAW, with 33% showing only psychological IPVAW. Moreover, the perpetration of this type of violence led to 47% of the victims needing medical attention, with the remaining 53% of the victims not being injured enough to need medical care. Finally, 90% of the IPVAW perpetrators sample did not present previous criminal records, leaving only 10% with a previous history of delinquency and/or violence.

### 3.1. Cognitive Assessment

Regarding working memory and attention assessment, significant group differences were found on Letters and numbers (Mann–Whitney U = −4.92, *p* < 0.001), spatial location (Mann–Whitney U = −2.70, *p* = 0.007), AST percentage of correct responses (Mann–Whitney U = −2.40, *p* = 0.016), and RVP (Mann–Whitney U = −3.06, *p* = 0.002). IPVAW perpetrators obtained worse performance on these tests than controls. (Table 2).

Regarding WCST performance, IPVAW perpetrators used more trials to complete the categories (Mann–Whitney U = −5.00, *p* < 0.001); they made more total and perseverative errors (Mann–Whitney U = −4.29, *p* < 0.001) and (Mann–Whitney U = −4.52, *p* < 0.001), respectively; and they completed fewer categories (Mann–Whitney U = −5.03, *p* < 0.001) than controls. 

With regard to the key test, differences were found between groups on the total score (Mann–Whitney U = −4.18, *p* < 0.001), with IPVAW perpetrators presenting lower scores than the controls. Nevertheless, we did not find any differences in total time to deliver and execute. 

The decision-making process subtests differed significantly between IPVAW perpetrators and controls on delay aversion (Mann–Whitney U = −1.92, *p* = 0.05), overall proportion of bets (Mann–Whitney U = −2.10, *p* = 0.035), and risk taking (Mann–Whitney U = −2.12, *p* = 0.033), with IPVAW perpetrators showing higher delay aversion, overall proportion of bets, and risk-taking scores than controls. 

### 3.2. Relationships Between Drug Use and Cognitive Performance in Both Groups

In the entire sample, the number of alcohol grams per week was related to worse cognitive performance. In fact, it was negatively associated with letters and numbers (*r* = −0.255, *p* < 0.01), spatial location (*r* = −0.287, *p* < 0.001), key test total score (*r* = −0.215, *p* < 0.05), and WCST number of categories completed (*r* = −0.272, *p* < 0.001). Moreover, it was positively related to WCST total trials (*r* = 0.211, *p* < 0.05), WCST number of errors (*r* = 0.262, *p* < 0.001), CGT overall proportion of bets (*r* = 0.265, *p* < 0.001), and CGT risk taking (*r* = 0.278, *p* < 0.001). 

In the entire sample, the number of cigarettes per week and/or number of years consuming cigarettes was unrelated to the cognitive variables.

Regarding cannabis consumption, the number of joints per week was negatively related to cognitive performance. In fact, it was associated with the key test total time (*r* = −0.217, *p* < 0.05). Furthermore, it was positively associated with CGT overall proportion of bets (*r* = 0.265, *p* < 0.01), and CGT risk taking (*r* = 0.281, *p* < 0.001). Moreover, the number of years consuming cannabis was negatively associated with CGT overall proportion of bets (*r* = 0.280, *p* < 0.001), and CGT risk taking (*r* = 0.264, *p* < 0.01).

Regarding cocaine misuse, the number of grams per week was associated with AST congruency (*r* = 0.434, *p* < 0.01), RVP (*r* = −0.393, *p* < 0.01). In other words, higher weekly cocaine misuse entailed worse cognitive performance. 

In the case of heroin misuse, as in previous cases, a higher number of years entailed worse cognitive performance. In this sense, significant associations were found between number of years consuming and spatial location (*r* = −0.244, *p* < 0.01) WCST number of categories completed (*r* = −0.261, *p* < 0.01), and CGT quality of decision making (*r* = −0.295, *p* < 0.001). 

Finally, with regard to MDMA consumption, the number of pills per week was associated with RVP (*r* = −0.336, *p* < 0.001). Furthermore, the number of years consuming was significantly related to WCST number of categories completed (*r* = −0.257, *p* < 0.01), spatial location (*r* = −0.250, *p* < 0.01), and CGT quality of decision making (*r* = −0.373, *p* < 0.001). This might mean that higher MDMA consumption entails worse cognitive performance.

Afterwards, we checked for the moderator role of ‘group’ in the previously mentioned relationships (see data analysis section). We ran hierarchical regression models, including ‘group’, ‘drug misuse’, and the ‘group x drug misuse’ interaction as independent variables and cognitive performance as dependent variable. Nevertheless, there was no significant moderator role of ‘group’ found in the relationship between drug misuse and cognitive performance. Hence, we cannot assume that the associations between those variables differ between IPVAW perpetrators and controls.

Exclusively for IPVAW perpetrators, we checked for the relationships between drug misuse (amount of weekly consumption and number of consecutive years consuming) with risk of reoffending, type of aggression, severity of injuries, and criminal history. 

Regarding risk of reoffending as assessed by interviewers, a significant relationship was found with number of years consuming alcohol (*r* = 0.291, *p* < 0.01) and the number of cigarettes per week (*r* = 0.349, *p* < 0.001).

With regard to the type of aggression, it was significantly associated with grams of cocaine per week (*r* = 0.485, *p* < 0.001).

Severity of injuries was associated with number of years consuming alcohol (*r* = −0.374, *p* < 0.001), grams of cocaine per week (*r* = −0.313, *p* < 0.001), and number of cigarettes per week (*r* = −0.412, *p* < 0.001).

Finally, previous criminal story was significantly associated with number of years consuming heroin (*r* = 0.287, *p* < 0.001), number of joints per week (*r* = 0.382, *p* < 0.001), number of years consuming cannabis (*r* = 0.307, *p* < 0.001) and the number of years consuming cocaine (*r* = 0.596, *p* < 0.001).

## 4. Discussion

Compared to the controls, IPVAW perpetrators showed considerably higher levels of drug misuse. Particularly, they presented higher amounts of weekly alcohol, cannabis, cocaine, and heroin misuse, that were sustained over the years than the controls. Regarding the cognitive variables, the IPVAW perpetrators’ performance was characterized by worse working memory and attention (sustained and switched); they also presented difficulties in planning and changing their decisions in a changing environment, and they made riskier decisions than controls. Moreover, the highest drug misuse sustained over time was related to the worst cognitive performance and the highest IPVAW severity. Lastly, alcohol, tobacco, and cocaine seemed to be related to IPVAW and risk of reoffending. Whereas, heroin, cannabis, and MDMA were related to the existence of a previous criminal record (delinquency without violence).

With regard to the first aim of our study, the results demonstrated that IPVAW perpetrators presented higher drug misuse (e.g., alcohol, cannabis, cocaine, and heroin) than controls, which is consistent with previous findings in this field of research [5,10,11,12]. Although, the most commonly used drug is alcohol, a large number of participants also consumed cannabis, cocaine, and heroin. Nevertheless, the controls consumed more nicotine and an equal number of MDMA pills every week, but for fewer years than IPVAW perpetrators. Even though batterers tend to start to consume drugs later, their drug intake, during the week, is clearly higher than controls. This result is especially interesting and calls for attention to the fact that weekly alcohol consumption by IPVAW perpetrators is higher than 50 g per week, being approximately 240 g per week. In fact, several studies have stated that a weekly consumption greater than this amount is extremely harmful to health, cognition, and/or behavioral regulation [40,41,42,43,44,45,46], even considering that Spain is a Mediterranean country with a permissive culture regarding alcohol consumption. In fact, the daily consumption of alcohol, such as a glass of wine or beer [47], is common. Thus, reducing alcohol levels, during the initial stages of treatment, is a priority.

Because most IPVAW perpetrators tend to consume alcohol, it is highly likely that some of these individuals combine alcohol with other drugs. It is common to combine these drugs, exponentially increasing their effects (i.e., alcohol with cocaine, alcohol with cocaine and cannabis), or to combat side effects caused by one of them [11,12]. Nevertheless, concurrent alcohol and cocaine misuse tends to be related to greater cognitive dysfunction, in comparison with individuals who only consume one of these substances [10,11,12,48]. In this line, our current data demonstrated that, alcohol, cannabis, and cocaine were associated with similarly altered cognitive functioning. Thus, it makes sense to imagine that concurrent misuse of these drugs might explain, at least in part, why IPVAW perpetrator groups presented slightly worse cognitive functioning than the controls. 

As previously stated, IPVAW perpetrators exhibited greater impairments in working memory, processing speed, attention, cognitive flexibility/set-shifting, planning, and decision-making processes than non-violent men [10,49,50,51,52,53,54,55]. Our results extend previous conclusions, that characterize IPVAW perpetrators as individuals with working memory difficulties, in sustaining and switching attention, as well as difficulties in planning and flexibility in changing their behavior in a demanding context. Additionally, they tend to make risky decisions about money and spend large amounts of money on gambling tasks. These results are interesting because recently published studies demonstrated that IPVAW perpetrators, who dropped out of interventions during the initial stages, had important cognitive deficits, the highest alcohol consumption, and the highest rate of reoffending [52,53,54]. Therefore, our data reinforce the need to develop an initial cognitive screening to assess the cognitive status before starting the intervention. This screening will make it possible to develop coadjutant cognitive rehabilitation programs for these cognitive dysfunctions/alterations, increasing treatment adherence and decreasing future risk of IPVAW reoffending [54,56,57,58]. 

As far as we know, there is a gap in the scientific literature in analyzing the patterns of drug misuse and their associations with cognitive performance in IPVAW perpetrators. Except for nicotine, the rest of the drugs presented the same association with cognitive performance. In other words, the highest weekly consumption, sustained over time, was related to the worst cognitive performance. Our data point out that, it is not only important to assess alcohol consumption, but also other drugs, whose effects might be equal to or worse than alcohol. In this regard, it is particularly important to assess drug misuse of convicted men during the initial stages of intervention, in order to decrease the risk of drop-out and reoffending. 

Finally, interesting associations were also found between drug misuse and severity of IPVAW perpetration. In fact, alcohol, nicotine, and cocaine seem to be related to IPVAW severity (type of aggression, severity of injuries, and risk of reoffending. Whereas, heroin, cannabis, and MDMA were related to the existence of a previous criminal history (delinquency without violence). Curiously, the most consumed drugs were those related to aggressive reactions, but the rest of the drugs were more associated with delinquency. These results reinforce the need for interventions that incorporate coadjutant interventions focusing on this need of aggressors. In this sense, incorporating coadjutant substance treatments to IPVAW interventions has been effective in considerably reducing the IPVAW risk of reoffending [59].

Despite our promising results, the limited sample size of the study, as well as the cross-sectional and non-experimental nature of our study, kept us from generalizing our results. Thus, future studies should attempt to replicate our findings in a longitudinal study, with a much larger sample size. Furthermore, because we did not provide standardized scores for each participant, we cannot assume that the IPVAW group could be qualified as individuals with a “deficit” and/or “impaired”. In fact, in order to confirm the existence of deficits, participants should score < 1.5 SD below the mean, compared to a normative group. Therefore, we can only conclude that IPVAW offenders’ performance was slightly worse than the controls. Furthermore, we would recommend that other samples of IPVAW perpetrators are included in future studies, for example incarcerated IPVAW perpetrators, in order to compare their characteristics, increasing this way the generality of our results. Moreover, it would be advisable to incorporate saliva, blood and/or urine analysis, which indicate current drug use. This, in combination with self-reports and psychotherapist’s experiences, might make it possible to confirm the existence of a drug use disorder. Additionally, future studies should attempt to incorporate other psychobiological correlates of drug misuse, such as electroencephalographic techniques, psychophysiological measurements, hormonal parameters, and genetic markers, among others. These measures would make it possible to increase the validity and interest of our study by increasing the number of variables analyzed. 

Our study reinforces the need to include additional screening measurements during the initial stages of intervention programs, that have been designed for male abusers. Moreover, we also targeted different cognitive domains for specific cognitive training interventions, designed to prevent violence recidivism, in the long-term, through their effects on emotional information processing and behavioral regulation. These novel data can help to improve our understanding of the factors involved in IPVAW perpetration and the risk of becoming violent. Greater knowledge about the relationships between these variables could be useful in developing intervention programs for IPVAW perpetrators. In fact, this study indicates several variables that could direct modulators of violence through their effects on emotional information processing and executive functioning. The skills in these areas could be strengthened by reducing drug misuse and providing specific neuropsychological rehabilitation programs as adjuvant treatments to psychotherapy. 

## Figures and Tables

**Table 1:** Mean ± SD of demographic and drug misuse for all groups (* *p* < 0.05, ** *p* < 0.01, *** *p* < 0.001).

			IPVAW (*n* = 63)	Controls (*n* = 39)
**Demographic variables**				
**Age (years)**			39.73 ± 10.72	41.72 ± 11.01
**Nationality**				
Spanish			73%	82%
Other nationalities			27%	18%
**Marital status**				
Single/Divorced/Widowed			64%	78%
Married			36%	22%
**Number of children**			1.11 ± 1.45	0.79 ± 0.95
**Level of education**				
Primary/lower secondary			57%	44%
Upper secondary/vocational training			43%	56%
**Employment status**				
Employed			59%	46%
Unemployed			41%	54%
**Drug misuse (weekly)**				
**Alcohol use**	**Yes**		80%	85%
	**No**		20%	15%
Number of grams per week ***			232.59 ± 425.11	49.37 ± 72.91
Number of years consuming *			16.94 ± 12.20	22.74 ± 10.75
Nicotine ***	**Yes**		67%	30%
	**No**		33%	70%
Number of cigarettes per week *			57.25 ± 68.03	28.70 ± 55.28
Number of years consuming **			2.69 ± 7.25	11.05 ± 14.49
Cannabis *	**Yes**		24%	5%
	**No**		76%	95%
Number of joints of cannabis per week ***			8.79 ± 16.15	1.49 ± 4.69
Number of years consuming			4.65 ± 8.23	2.56 ± 5.52
**Cocaine**	**Yes**		7%	7%
	**No**		93%	93%
Number of grams per week *			0.85 ± 2.39	0.23 ± 0.79
Number of years consuming **			3.82 ± 6.63	1.13 ± 2.81
Heroin *	**Yes**		5%	0%
	**No**		95%	100%
Number of grams per week **			0.60 ± 4.44	-
Number of years consuming **			0.39 ± 2.36	-
**MDMA**	**Yes**		6%	5%
	**No**		94%	95%
Number of pills per week			0.20 ± 1.08	0.06 ± 0.32
Number of years consuming			0.45 ± 2.45	0.41 ± 1.83
**Androgenic steroids**	**Yes**	**No**	-	-
Number of years consuming			-	-

**Table 2:** Mean ± SD of Memory tests of all groups (**p < 0.01, ** *p* < 0.01, ***p < 0.001).

	IPVAW (*n* = 63)	Controls (*n* = 39)
**Working memory (verbal and visuo-spatial)**		
Letter and number *** Spatial location **	7.63 ± 2.92 15.47 ± 3.67	10.85 ± 2.78 17.62 ± 3.38
**Sustained attention, set-switching and speed processing**		
RVP * AST congruency AST switch cost AST percentage of correct responses ***	0.88 ± 0.06 106.65 ± 82.88 −99.67 ± 113.10 84.34 ± 17.15	0.91 ± 0.08 92.18 ± 81.56 −142.44 ± 116.86 92.03 ± 6.73
**Executive functions**		
**Planning**		
Total time (planning + execution) Total score ***	36.17 ± 20.70 11.21 ± 3.58	47.59 ± 34.58 7.84 ± 3.59
**Cognitive flexibility**		
Total trials *** Total errors *** Perseverative mistakes *** Completed categories ***	116.90 ± 19.92 50.03 ± 25.33 26.87 ± 15.10 3.51 ± 1.91	93.09 ± 20.99 26.07 ± 21.44 13.70 ± 13.46 5.34 ± 1.54
**Decision making**		
Deliberation time (ms) Overall proportion bet * Delay aversion * Quality of decision making Risk adjustment Risk taking *	3081.64 ± 1760.50 0.58 ± 0.16 0.21 ± 0.24 0.83 ± 0.14 0.75 ± 1.02 0.62 ± 0.16	2587.29 ± 801.52 0.51 ± 0.18 0.12 ± 0.19 0.85 ± 0.15 0.54 ± 0.17 19.39 ± 3.84
